# Umbilical cord blood bilirubins, gestational age, and maternal race predict neonatal hyperbilirubinemia

**DOI:** 10.1371/journal.pone.0197888

**Published:** 2018-06-01

**Authors:** Adrian Castillo, Tristan R. Grogan, Grace H. Wegrzyn, Karrie V. Ly, Valencia P. Walker, Kara L. Calkins

**Affiliations:** 1 Department of Medicine, Stanford University Medical Center, Palo Alto, California, United States of America; 2 David Geffen School of Medicine, University of California Los Angeles, Los Angeles, California, United States of America; 3 Department of Medicine, Statistics Core, David Geffen School of Medicine, University of California Los Angeles, Los Angeles, California, United States of America; 4 Loyola University of Chicago Stritch School of Medicine, Chicago, Illinois, United States of America; 5 Department of Pediatrics, Division of Neonatology, David Geffen School of Medicine, University of California Los Angeles, UCLA Mattel Children’s Hospital, Los Angeles, California, United States of America; Centre Hospitalier Universitaire Vaudois, FRANCE

## Abstract

**Objective:**

No validated biomarker at birth exists to predict which newborns will develop severe hyperbilirubinemia. This study’s primary aim was to build and validate a prediction model for severe hyperbilirubinemia using umbilical cord blood bilirubins (CBB) and risk factors at birth in neonates at risk for maternal-fetal blood group incompatibility. This study’s secondary aim was to compare the accuracy of CBB to the direct antigen titer.

**Methods:**

Inclusion criteria for this prospective cohort study included: ≥35 weeks gestational age, mother with blood type O and/or Rh negative or positive antibody screen, and <24 hours of age. The primary outcome was severe hyperbilirubinemia, defined as phototherapy during the initial hospital stay. Secondary outcomes were a total serum bilirubin concentration >95^th^ and >75^th^ percentile during the initial hospital stay. The predictive performance and accuracy of the two tests (CBB and direct antigen titer) for each outcome was assessed using area under a receiver-operating characteristic curve (AUC), sensitivity, and specificity.

**Results:**

When compared to neonates who did not receive phototherapy (n = 463), neonates who received phototherapy (n = 36) had a greater mean CBB ± standard deviation (2.5 ± 0.7 vs. 1.6 ± 0.4 mg/dL, p<0.001). For every 0.3 mg/dL increase in CBB, a neonate was 3.20 (95% confidence interval, 2.31–4.45), 2.10 (1.63–2.70), and 3.12 (2.44–3.99) times more likely to receive phototherapy or have a total serum bilirubin concentration >95^th^ and >75^th^ percentile, respectively. The AUC ± standard error (95% confidence interval) for CBB for phototherapy and a total serum bilirubin concentration >95^th^ and >75^th^ percentile was 0.89 ± 0.03 (0.82–0.95), 0.81 ± 0.04 (0.73–0.90), and 0.84 ± 0.02 (0.80–0.89), respectively. However, the AUC for gestational age and maternal Asian race for these outcomes was only 0.55 ± 0.05 (0.45–0.66), 0.66 ± 0.05 (0.56–0.76), and 0.57 ± 0.04 (0.05–0.64), respectively. When the CBB was combined with gestational age and maternal Asian race, the AUC for a total serum bilirubin concentration >95^th^ percentile improved to 0.87 ± 0.03 (0.81–0.92) (p = 0.034 vs. the model with CBB only and p<0.001 vs. the model with clinical risk factors only). In a sub-group of subjects (n = 189), the AUC for the direct antigen titer for phototherapy was 0.64 ± 0.06 (0.52–0.77) with a 52% sensitivity and 77% specificity. In contrast, a CBB cut-point of 1.85 mg/dL was 92% sensitive and 70% specific for phototherapy with an AUC of 0.87 ± 0.04 (0.80–0.95).

**Conclusion:**

CBB, in combination with gestational age and maternal race, may be a useful, non-invasive test to predict shortly after birth which neonates will develop severe hyperbilirubinemia.

## Introduction

Neonatal hyperbilirubinemia (HB) is a benign, transient phenomenon that occurs in most neonates. During the first week of life, an increase in bilirubin production and decrease in bilirubin elimination cause total serum bilirubin (TSB) concentrations to rise [1, 2]. Thereafter, the liver efficiently conjugates and eliminates bilirubin, and TSB concentrations normalize. During this time, some newborns develop severe HB requiring treatment with phototherapy (PT). Extremely high and rapidly rising TSB concentrations can cause kernicterus, which can result in death and cerebral palsy. The American Academy of Pediatrics (AAP) recommends assessing newborns for HB prior to nursery discharge by performing a risk assessment and measuring a bilirubin concentration [3]. Maternal-fetal blood group incompatibility can cause hemolytic disease of the fetus and newborn (HDFN), a risk factor for early-onset HB and bilirubin-induced neurotoxicity [3]. Common causes of HDFN include ABO and Rhesus (Rh) incompatibility. In cases of HDFN, maternal immunoglobulin G (IgG) antibodies cross the placenta and hemolyze fetal and neonatal red blood cells causing severe HB.

Despite widespread screening, severe HB continues to be a health problem [1, 3–5]. While total bilirubin concentrations are traditionally measured to risk stratify newborns and determine PT treatment, clinicians lack a test *at birth* to predict HB. As early as the 1970s, there was some evidence that bilirubins obtained from umbilical cord blood could predict severe HB [6]. While TSB concentrations represent neonatal bilirubin metabolism, umbilical cord blood bilirubin (CBB) concentrations represent fetal bilirubin metabolism. In utero, the placenta plays a major role in bilirubin excretion, while the liver’s capacity to metabolize bilirubin is limited [7]. Hence, if hemolysis is occurring in utero, CBB concentrations would be predicted to rise [6, 8–14]. Studies have investigated the utility of CBB, but results are conflicting [6, 8–14]. Some studies were retrospective [7]. In addition, many studies had small sample sizes, did not uniformly measure total bilirubin concentrations, and neglected well-established HB risk factors [6, 8–14].

Considering the potential utility of CBB and the above stated limitations, we designed a prospective cohort study that targeted neonates at risk for HDFN secondary to maternal-fetal blood group incompatibility. The study’s primary objective was to build a prediction model for severe HB using CBB and well-known clinical risk factors present at birth in a diverse population in the United States. The study’s secondary objective was to compare CBB to a commonly used test to diagnosis HDFN, the direct antigen titer (DAT), or Coombs’ test [3]. We hypothesized that: *1)* A model that combined CBB with well-known clinical risk factors at birth in comparison to a model with clinical risk factors alone would have a greater predictive accuracy for severe HB, and *2)* CBB, in comparison to DAT, would have a greater sensitivity and specificity for severe HB. These hypotheses are based on previous studies that have demonstrated that neonates with severe HB, blood group incompatibility, and HDFN have greater CBB concentrations in comparison to neonates without these risk factors [6, 8–14].

## Methods and materials

### Study design

To test our hypotheses, we enrolled neonates at risk for maternal-fetal blood group incompatibility and prospectively followed subjects until nursery discharge. Subjects were recruited from the newborn nursery at Mattel Children’s Hospital (University of California, Los Angeles, Los Angeles, California, United States). Inclusion criteria included gestational age ≥35 weeks, maternal blood type O and/or Rh negative or a positive antibody screen, and admission to the newborn nursery after birth. Exclusion criteria included major congenital anomalies, congenital infections, liver disorders, and maternal history of hepatitis. At our institution, CBB concentrations and neonatal blood types from umbilical cord blood are routinely performed if there is a maternal history of blood type O, Rh-negative status, or a positive antibody screen. If blood type incompatibility is present, a DAT is performed using umbilical cord blood. CBB and DAT results are available to the primary medical team shortly after birth. Other screening labs for hemolysis, such as reticulocyte count, peripheral smear, and hematocrit, are not routinely performed in our newborn nursery.

This study’s primary outcome was severe HB, defined as PT during the initial hospital stay. In general, the primary medical team followed the AAP Clinical Guidelines for HB screening and PT treatment [3]. HB interventions were at the discretion of the medical team. To determine if PT was clinically indicated, the research team used the AAP hour specific nomogram [3]. The AAP recommends considering PT if the TSB concentration exceeds the value indicated by one of three curves on the nomogram [3]. The AAP also states that it is an option to start PT at TSB concentrations 2–3 mg/dL below the curve [3]. In order to determine which curve to use, the research team categorized subjects as: *1)* low risk (>38 weeks gestation and well), *2)* medium risk (>38 weeks gestation with risk factors or 35–37 6/7 weeks gestation), or *3)* high risk (35–37 6/7 weeks gestation with risk factors) [3]. Risk factors included HDFN (TSB concentration > 95^th^ percentile and a positive DAT and/or elevated reticulocyte count), sepsis, lethargy, hypoalbuminemia, acidosis, and temperature instability [3]. For this study, PT was considered clinically indicated if the subject’s TSB concentration was greater than the TSB concentration that was 3 mg/dL below the subject’s curve.

Secondary outcomes for this study included any TSB concentration >95^th^ and >75^th^ percentile according to the AAP nomogram during the initial hospital stay [3]. Per our nursery’s guidelines, at least one TSB is performed prior to discharge. Generally, a heel-stick is performed to measure TSB at the same time as the newborn screen. TSB concentrations are measured by the diazo method (Roche Cobas 8000 chemistry analyzer, Diamond Diagnostics, MA).

Data were prospectively collected. This study was approved by the institutional review board at the University of California, Los Angeles, and conducted according to the principles set forth in the Declaration of Helsinki. Informed consent and parental permission was obtained from all subjects.

### Statistical analysis

Prior to the start of the study, the frequency of the primary outcome (PT) in neonates at risk for maternal-fetal blood group incompatibility was 15%. In neonates not at risk for maternal-fetal blood group incompatibility, the frequency was 5%. From our retrospective case control study, we ran a logistic regression model with established clinical risk factors at birth for severe HB [9]. These risk factors, or clinical variables, were specified *a priori* and included sex, gestational age, birth weight, maternal Asian race, and gestational diabetes [3]. This model, referred to as the clinical variable model, yielded an area under a receiver-operator characteristic curve (AUC) of 0.55. Given a sample size of 500 (approximately 50 subjects treated with PT and 450 subjects not treated with PT), the study would have 90% power to detect a delta AUC of 0.15 assuming CBB increased the AUC from 0.55 to 0.70. We believed an AUC of 0.70 was feasible to attain since our previous study found CBB alone to have an AUC of 0.87 [9].

Our final predictive model would include CBB and clinical variables, referred to as the CBB and clinical variable model. Clinical variables (and CBB) were included in these models if they were selected across any of the five variable selection algorithms (three stepwise algorithms: forward/backward/both, best subsets, and the LASSO) among the study’s three outcomes (PT and TSB concentration >95^th^ and >75^th^ percentile). We used this rationale because we wanted our models to be consistent regardless of the outcome, and to err on the side of caution. Across the three outcomes and five variable selection techniques, three variables, which included CBB, gestational age, and maternal Asian race, were consistently selected. Therefore, the clinical variable model included terms for gestational age and maternal Asian race. The clinical variable model was then compared to our CBB model and CBB and clinical variable model by examining the predictive performance, or delta AUC, for each outcome and comparing the AUCs using the DeLong test [15].

To validate our predictive models and obtain approximations for how well these models generalize to external samples, the bias-adjusted AUC was assessed on all 3 multivariate models, by randomly splitting the dataset into 2/3 training and 1/3 test sets. We then fit the models built from the training data and assessed their fit on test data (1,000 times). We computed the average AUC across 1,000 simulations as well as proposed confidence intervals (CI).

In order to assess the relative sensitivity and specificity of DAT against CBB, we performed a subgroup analysis on subjects who had both tests. A CBB cut-point for comparison was chosen based on the *a prioir* hypothesis that to be clinically useful, the test should have at least 90% sensitivity.

Clinical characteristics and study variables were compared between the PT and non-PT groups using t-tests, or chi-square or Fisher’s exact test. Statistical analyses were performed using R V3.1.2 (Vienna, AU) and SPSS V24 (Armonk, NY) [16, 17]. P-values <0.05 were considered statistically significant. Data are represented as means (± standard deviations). All AUCs are represented with standard errors (95% CI).

## Results

From June 2014 to July 2016, 1257 newborns were eligible, 503 subjects were enrolled, and 499 subjects completed the study. The mean CBB (± standard deviation) for the entire cohort was 1.7 ± 0.5 mg/dL (range 0.2–5.1 mg/dL, 95^th^ percentile 2.4 mg/dL). Seventy six percent (n = 378) of the mothers identified as Caucasian, 16% (n = 82) identified as Asian, and 5% (n = 26) identified as African American. The remaining mothers identified as other. Gestational age was 39.5 ± 1.2 weeks, and birth weight was 3.4 ± 0.4 kg.

Ninety percent of mothers had blood type O (n = 447), 19% were Rh negative (n = 94), and 13% were antibody positive (n = 63). Thirty eight percent (n = 189) of the neonates had maternal-fetal blood group incompatibility. CBBs were greater in neonates with ABO blood group incompatibility vs. neonates without ABO blood group incompatibility (1.8 ± 0.5 vs. 1.6 ± 0.5 mg/dL, p = 0.002). CBBs were not significantly different when neonates with Rh incompatibility were compared to neonates without Rh incompatibility (1.7 ± 0.4 vs. 1.7 ± 0.5, p = 0.82).

7.2 percent (n = 36) were treated with PT. The remaining subjects (n = 463) did not receive PT. PT was indicated in 66.7% (n = 24) of subjects who were treated with PT. Table 1 shows the demographics, baseline characteristics, and data collected during the subjects’ nursery stay for subjects who received PT and subjects who did not receive PT. When compared to subjects who did not receive PT, subjects who received PT had significantly greater CBB concentrations (2.5 ± 0.7 vs. 1.6 ± 0.4 mg/dL, p<0.001), were more likely to have maternal-fetal blood group incompatibility (72.2% vs. 35.2%, p<0.001) and a TSB concentration >95^th^ (47.2% vs. 2.4%, p<0.001) and >75^th^ percentile (97.2% vs. 13.8%, p<0.001) (Table 1). CBB concentrations were also greater in neonates with a TSB concentration >95^th^ (2.4 ± 0.8 vs. 1.7 ± 0.4 mg/dL, p<0.001) and >75^th^ percentile (2.2 ± 0.6 vs. 1.6 ± 0.3 mg/dL, p<0.001).

**Table 1:** Baseline characteristics and outcomes.

	+ PT (n = 36)	- PT (n = 463)	P-Value
Maternal Characteristics			
Age, years	32.8 ± 4.8	32.1 ± 5.1	0.40
Race			0.65
African-American	1 (2.8)	25 (5.4)	
Asian	8 (22.2)	74 (16.0)	
White	26 (72.2)	352 (76.0)	
More than one race or other	1 (2.8)	12 (2.6)	
Hispanic	7(19.4)	139 (30.0)	0.25
Gravida	2.3 ± 1.5	2.3 ± 1.4	0.75
Para	1.7 ± 1.0	1.8 ± 1.0	0.62
Gestational diabetes mellitus	1 (2.8)	35 (7.6)	0.50
Positive maternal antibody screen	5 (13.9)	58 (12.5)	0.80
Blood type			0.92
O	33 (91.7)	414 (89.4)	
A	3 (8.3)	33 (7.1)	
B	0 (0.0)	11 (2.4)	
AB	0 (0.0)	5 (1.1)	
Rh negative	8 (22.2)	86 (18.6)	0.75
Rhogam given prior to delivery	5 (62.5)	54 (62.8)	1.00
Neonatal Characteristics			
Gestational age, weeks	39.4 ± 1.6	39.5 ± 1.2	0.87
Birth weight, kg	3.4 ± 0.4	3.4 ± 0.4	0.84
Male sex	20 (55.6)	242 (52.3)	0.84
Vaginal delivery	23(63.9)	327 (70.6)	0.51
Apgar score 5 min	8.8 ± 0.4	8.9 ± 0.5	0.25
Blood type, n (%)			<0.001
O	10 (27.8)	310 (67.0)	
A	13 (36.1)	111 (24.0)	
B	13 (36.1)	35 (7.6)	
AB	0 (0.0)	6 (1.3)	
Unknown	0 (0.0)	1 (0.2)	
Blood group incompatibility	26 (72.2)	163 (35.2)	<0.001
Positive DAT	13 (52.0)^a^	38 (23.2)^b^	0.003
Family history of glucose-6-phosphate dehydrogenase deficiency	1 (2.8)	2 (0.4)	0.19
Sibling who required PT	9 (40.9)^c^	34 (11.8)^d^	0.001
Early onset sepsis^e^	1 (2.8)	0 (0.0)	0.07
Weight loss % at discharge	-5.7 ± 3.5	-5.1 ± 2.9	0.24
Exclusive breastfeeding	15 (41.7)	331 (71.5)	<0.001
Study Outcomes			
TSB >75^th^ percentile	35 (97.2)	64 (13.8)	<0.001
TSB >95^th^ percentile	17 (47.2)	11 (2.4)	<0.001
Cord blood bilirubin, mg/dL	2.5 ± 0.7	1.6 ± 0.4	<0.001
Maximum TSB, mg/dL	11.1 ± 3.0	6.2 ± 2.1	<0.001
Length of stay, hours	67.6 ± 23.2	51.5 ± 22.2	<0.001

Data are represented as means (± standard deviations) or n (%).

^a^n = 25.

^b^n = 164.

^c^n = 22.

^d^n = 287.

^e^Early onset sepsis was defined as a positive blood culture and antibiotic treatment > 5 days.

### Predictive value of umbilical cord blood bilirubin

**Fig 1:**
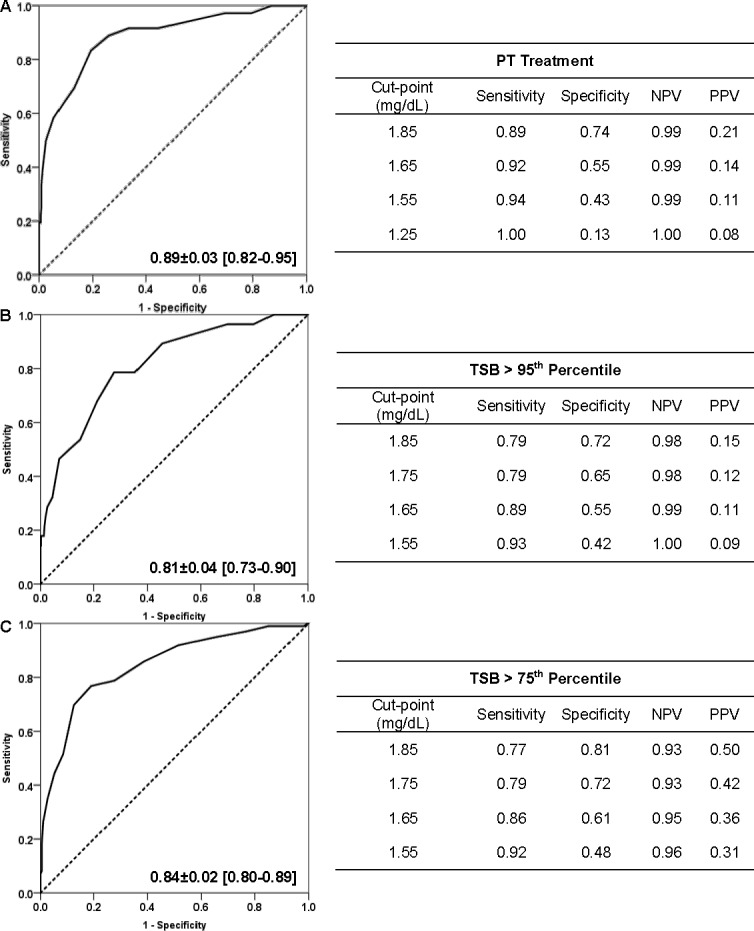
Receiver-operator characteristic curve and corresponding AUC±SE (95% CI) statistics for CBB predictive value for (A) PT, (B) TSB > 95th percentile, and (C) TSB > 75th percentile. Tables to the right show specific cut-points and the respective sensitivity, specificity, negative predictive value (NPV), and positive predictive value (PPV).

**Fig 2:**
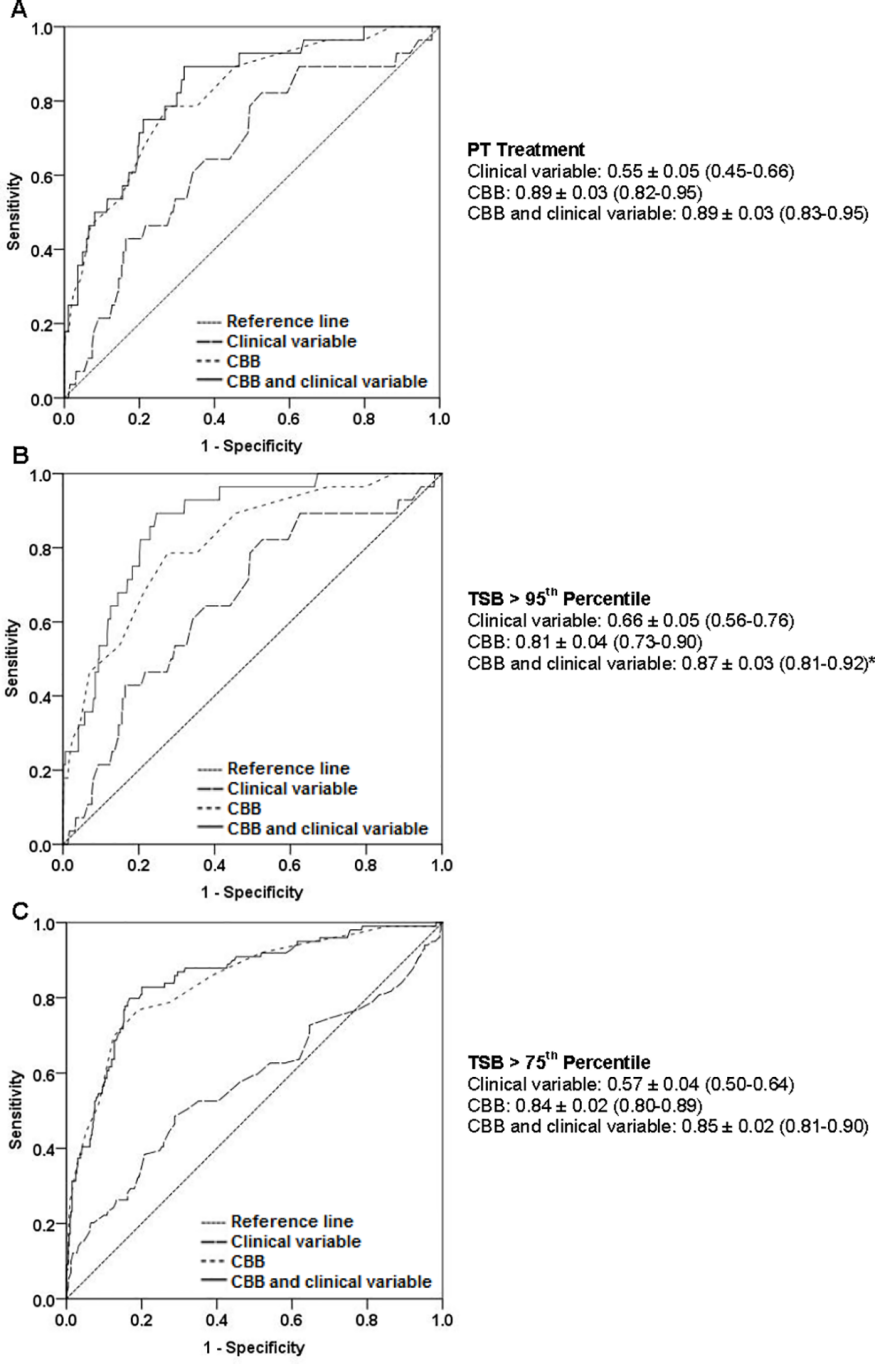
Receiver-operator characteristic curve and corresponding AUC±SE (95% CI) values for (A) PT, (B) TSB>95^th^ percentile, and (C) TSB>75^th^ percentile for the three models (clinical variable, CBB, and CBB and clinical variable model). The clinical variables include gestational age and maternal Asian race. *delta AUC = 0.07, p = 0.034.

The AUC ± standard error (95% CI) for CBB for PT and TSB concentration >95^th^ and >75^th^ percentile was 0.89 ± 0.03 (0.82–0.95), 0.81 ± 0.04 (0.73–0.90), 0.84 ± 0.02 (0.80–0.89), respectively (Fig 1). Bias adjusted AUCs were also calculated and results were identical (data not provided). When compared to the clinical variable model, the CBB model and CBB and clinical variable model performed significantly better (delta AUC of 0.15–0.34, p<0.05 for all) (Fig 2). For every 0.3 mg/dL increase in CBB, a neonate is 3.20 (95% CI 2.31–4.45), 2.10 (1.63–2.70), and 3.12 (2.44–3.99) times more likely to receive PT or have a TSB concentration >95^th^ and >75^th^ percentile, respectively. Neonates born to Asian mothers had a higher odds of having a TSB concentration >95^th^ (odds ratio 3.08, 95% CI 1.37–6.94) and > 75^th^ percentile (odds ratio 2.19, 95% CI 1.29–3.71) (Table 2).

**Table 2:** Univariate and multivariate analysis for phototherapy treatment, and TSB > 95^th^ and TSB > 75^th^ percentile.

	Phototherapy		TSB > 95^th^ Percentile		TSB > 75^th^ Percentile	
	Odds Ratio	95% CI	Odds Ratio	95% CI	Odds Ratio	95% CI
Univariate Analysis						
Birth weight, kg	1.08	0.50–2.32	1.00	0.42–2.38	1.02	0.62–1.67
Gestational age, weeks	0.98	0.76–1.26	0.91	0.71–1.17	1.08	0.91–1.29
Gestational diabetes	0.35	0.05–2.62	1.59	0.46–5.40	1.38	0.63–3.03
Male sex	1.14	0.58–2.26	1.43	0.65–3.11	1.05	0.68–1.64
Caucasian race	0.82	0.38–1.75	0.47	0.21–1.04	0.72	0.44–1.18
Asian race	1.50	0.66–3.42	3.08	1.37–6.94^†^	2.19	1.29–3.71^‡^
CBB, 0.3 mg/dL	3.20	2.31–4.45*	2.10	1.63–2.70*	3.12	2.44–3.99*
Multivariate Analysis						
Gestational age, weeks	0.80	0.64–1.00^†^	0.77	0.61–0.98^†^	0.94	0.78–1.13
Asian race	1.35	0.49–3.74	3.51	1.40–8.79^†^	2.20	1.16–4.15^†^
CBB, 0.3 mg/dL	3.36	2.40–4.69*	2.21	1.70–2.89*	3.15	2.45–4.04*

TSB, total serum bilirubin. CBB, cord blood bilirubin.

*p < 0.001

^†^p < 0.05

^‡^p < 0.10.

When CBB was combined with gestational age and maternal Asian race, the AUC for PT and TSB concentration >95^th^ and >75^th^ percentile were 0.89 ± 0.03 (0.83–0.95), 0.87 ± 0.03 (0.81–0.92), and 0.85 ± 0.02 (0.81–0.90), respectively (Fig 2). When the CBB model was compared to the CBB and clinical variable model, for all three outcomes, the CBB and clinical variable model performed significantly better than the CBB model for TSB concentration >95^th^ percentile (delta AUC of 0.07, p = 0.034). There was no difference when the AUCs were compared for the CBB vs. CBB and clinical variable model for the other two outcomes, PT (delta AUC of 0, p = 0.445) and TSB concentration >75^th^ percentile (delta AUC of 0.1, p = 0.114) (Fig 2).

### Accuracy of CBB vs. direct antigen test

CBB was greater in neonates with a positive DAT vs. neonates with a negative DAT (2.0 ± 0.6 vs. 1.7 ± 0.4 mg/dL, p<0.001). Fifty two percent (n = 13) of subjects who received PT had a positive DAT. In contrast, only 23.2% (n = 38) of subjects who did not receive PT had a positive DAT (p = 0.003) (Table 1). Subjects treated with PT and who had a positive DAT had a greater CBB concentration vs. untreated subjects with a positive DAT (2.6 ± 0.7 vs. 1.7 ± 0.4 mg/dL, p<0.001).

We performed a sub-group analysis (n = 189) to determine the predictive value and accuracy of DAT in comparison to CBB. The AUC for DAT for PT was 0.64 ± 0.06 (0.52–0.77) with a 52% sensitivity and 77% specificity. In this same subset of patients, a CBB cut-point of 1.85 mg/dL was 92% sensitive and 70% specific for PT. A CBB cut-point of 1.55 mg/d was 96% sensitive and 39% specific. The AUC for DAT for TSB concentration >95^th^ and >75^th^ percentile was 0.66 ± 0.07 (0.52–0.79) and 0.59 ± 0.05 (0.50–0.69), respectively. In contrast, the AUC for CBB for PT and TSB concentration >95^th^ and >75^th^ percentile was 0.87 ± 0.04 (0.80–0.95), 0.79 ± 0.05 (CI 0.70–0.89), and 0.88 ± 0.03 (0.82–0.94), respectively.

## Discussion

Jaundice remains the number one reason for hospital admission in the first week of life, and cases of kernicterus, which are 100% preventable, continue to be reported [1, 3–5, 18, 19]. In order to solve this problem, additional HB prediction tools are needed. Ideally, these tools would be practical, accurate, inexpensive, non-invasive, and would quickly predict which neonates are at high risk for severe HB. In this study, neonates who had severe HB had greater CBB concentrations than neonates who do not develop severe HB. Moreover, as hypothesized, the prediction model, CBB alone or in combination with clinical risk factors, performed significantly better than the clinical model alone for all three study outcomes (PT and TSB concentration >95^th^ and >75^th^ percentile). When CBB was combined with gestational age and maternal Asian race, the predictive value was significantly improved for the outcome TSB concentration >95^th^ percentile. While the addition of these two clinical risk factors did not improve the model’s predictive value for the other outcomes, we elected to include these terms in our final model for consistency.

TSB remains the gold standard to risk stratify and diagnosis severe HB [3]. While CBB will not eliminate TSB screening and testing, CBB may serve as an early biomarker and adjunct to TSB and transcutaneous bilirubin measurements. This may be relevant since nursery length of stay has decreased in recent years [20]. CBB may also help decrease the number of TSB tests performed and, at the same time, ensure that neonates at risk for severe HB are screened and treated in a timely manner.

CBBs are easily obtained from umbilical cord blood and have a short turnaround time. In a retrospective study of 12,993 Belgian neonates, a CBB of 1.98 mg/dL had a sensitivity at 71% with a specificity of 77% for a TSB concentration >95^th^ percentile [13]. In our study, a slightly lower CBB cut-off value of 1.85 mg/dL had a greater sensitivity at 79% and lower specificity at 72%. The negative predictive values (98–99%) and AUCs (0.81–0.82) were similar, and both studies had low positive predictive values [13]. In subgroup analysis of subjects with a positive DAT in this study, the sensitivity and specificity improved to 92% and 70%, respectively. The strength of CBB lies in its high sensitivity and negative predictive value. In other words, CBB will identify most infants who will develop severe HB at the expense of some false positives. As a result, clinicians can be re-assured that most neonates with a CBB less than 1.85 mg/dL are unlikely to develop severe HB.

In comparison to the Belgian and other studies, our study has advantages. First, our study was prospective [6, 8–14]. Second, our study was conducted over a short period of time (2014–2016) [6, 8–14]. As a result, we speculate that healthcare variation was minimal. Third, we enrolled a racially and ethnically diverse group of neonates [6, 8, 10–14]. However, these results may not be applicable to populations outside the United States. Fourth, all neonates in our study had a TSB. In contrast, in most studies, TSB concentrations were only measured in small sub-groups [6, 8, 10, 13]. Last, we developed and validated multivariate predictive models that combined CBB with maternal Asian race and gestational age for the prediction of three clinically relevant outcomes (PT, and TSB concentration >95^th^ and >75^th^ percentile) [3].

In our study, the AUC for TSB concentration >95^th^ percentile improved with the addition of maternal Asian race and gestational age. East Asian neonates have a higher risk for severe HB compared to non-East Asian neonates secondary to uridine diphosphoglucuronate glucuronosyltransferase mutations [3, 21, 22]. Premature neonates are also at high risk for severe HB due to an increase in enterohepatic circulation and decrease in bilirubin conjugation. In a study by Bhutani *et al*, TSB and transcutaneous bilirubin when combined with gestational age alone or gestational age and other clinical variables had a high predictive value for post-discharge PT (AUC 0.95 ± 0.2 (95% CI 0.93–0.97) and AUC 0.93 (95% CI 0.88–0.97)) [2]. In our study, the AUC for the CBB model and CBB and clinical variables model for PT treatment in the nursery was 0.89. While this AUC is slightly lower, advantages of this prediction model is that it does not involve a blood test and clinicians can identify high risk infants shortly after birth. Further research is warranted to determine if these variables can predict hospital re-admission for severe HB.

DAT results were not included in our final predictive model for several reasons. Not all infants had a DAT performed, and this test would add an additional expense. In previous studies, DAT has been shown to have a low positive predictive value for HDFN and PT [23, 24]. In this study, the sensitivity, specificity, and AUC for CBB was significantly greater than the sensitivity, specificity, and AUC for DAT for all three study outcomes. These results are similar to published studies that demonstrate a variable sensitivity of 53–85% [24, 25]. DAT’s low sensitivity is most likely because the test detects the presence of IgG antibodies on the red blood cell, not the antibody’s hemolytic potential.

The PT prescription rate in this study (6.7%) is similar to rates measured by other studies [18, 26]. Approximately 31% of subjects who received PT did not meet criteria for PT. We elected not to exclude these subjects from our model since this reflects clinical practice. We speculate that some clinicians may “over-prescribe” PT in an attempt to prevent severe HB as an outpatient [27].

Our study has limitations. This is a single site study, and further validation in a larger, multi-site study is warranted. Also, CBB results were not masked, and this may have biased the primary outcome (PT), which was determined by a physician. However, our secondary outcomes were not biased. While the AUC for CBB for PT was greater, the AUC for TSB concentration > 95^th^ and 75^th^ percentile when combined with gestational age and Asian race approached similar values. Interestingly, in this study, we would have anticipated that neonates with Rh incompatibility vs. neonates without Rh incompatibility would have greater CBBs [9]. However, CBBs were comparable between the two groups. It remains unclear if this finding is due to chance, sample size, or maternal treatment with Rhogam.

In conclusion, in this study, we developed a simple model using CBB, a non-invasive test, and gestational age and maternal Asian race, two clinical risk factors easily obtained from the medical chart, to predict severe HB during a newborn’s nursery stay (S1 File). Further investigation is warranted to determine if CBB can predict re-admission for HB, the utility of CBB in populations not at risk for HDFN, and the predictive value of CBB when combined with other non-invasive tests or HB risk factors that unfold during the nursery stay.

## Supporting information

S1 File Supplemental file. (XLSX)
